# A Study on Doctors' Perspective on PNDT Act

**DOI:** 10.4103/0970-0218.51219

**Published:** 2009-04

**Authors:** KM Dhaduk, DV Parmar, BS Yadav, Sudha Yadav

**Affiliations:** Department of Community Medicine, Shri M.P.Shah Medical College, Jamnagar, Gujarat, India

## Introduction

The Pre-Natal Diagnostic Techniques (PNDT) Act(1) was implemented in 1996 in view of the falling gender ratio, because of the misuse of sonography machines for prenatal gender determination by doctors. Not much data are available on the perception of the doctors on the PNDT Act. With this background in mind, the present study was undertaken.

## Materials and Methods

All obstetricians and radiologists who are major stakeholders in the implementation of the Pre-Conception and Pre-Natal Diagnostic Techniques (Prohibition of Sex Selection) Act were interviewed in Jamnagar. Out of 38 obstetricians and 13 radiologists, 26 obstetricians and 8 radiologists agreed to participate. In this study, the participant doctors were given pretested and structured proforma to give their opinion on various aspects related to the PNDT Act, viz. difficulties faced, penalties for violation, punishment for violation, genuineness of information (Forms F and G), repercussions of the PNDT Act, demand for gender determination, and suitable amendments in the PNDT Act between July 2006 and September 2006. For analysis of data, SPSS Version 11.0 was used.

## Results

Only 5.91% of the doctors felt that the PNDT Act is the only tool for improving the gender ratio. As many as 79.41% of the doctors were of the opinion that the PNDT Act is not the only tool to improve the gender ratio while 14.7% had no opinion.

About a quarter (26.55%) of the doctors were of the view that penalties for violating the PNDT Act are very heavy while three-quarters (73.45%) did not feel so.

A total of 67.6% of the doctors were of the view that publicity through the media of court cases related to breaches of the PNDT Act by doctors is beneficial for improving the gender ratio as it will act as a deterent against flouting the provision of the PNDT Act by doctors. A total of 32.41% of the doctors did not feel the same way.

On inquiring regarding completing forms F and G genuinely and completing it with true information, about half (55.9%) of the doctors stated that they completed these forms genuinely and with correct information; 2.9% stated that the information completed was absolutely false and 41.2% were not sure.

With regard to the impact of the PNDT Act on the future progress of the invention related to use of ultrasound technique in medical sciences; as many as 41.2% of doctors felt that the PNDT Act can hamper the future course of medical invention, 44.1% of the doctors did not think so, and 14.7% of the doctors did not know.

When asked about the demand from doctors for gender determination by patients in the Outpatient Department, 97.1% confirmed that there is such a demand from patients or her family.

A total of 26.4% of the doctors were in favor of dropping the provision of registration of a sonography machine on a periodical basis i.e., every 3 years and felt that it should only be a one-time registration. Similarly, these same doctors also felt that if changing the place of the clinic, a clause of fresh registration for the same sonography machine should be omitted from the PNDT Act. Of the remaining doctors, 58.82% had no such opinion and 14.71% did not give any response.

A total of 73.5% of the doctors felt that along with the doctor, the patient and family members involved should also be punished for violating the PNDT Act. Another 14.7% of doctors felt that both the doctor and the patient should be punished, 8.8% felt only the mother should be punished, and 2.9% felt that only the family member should be punished [Table 1].

**Table 1:** To whom punishment should be given for violation of the PNDT Act

Doctors' views	No. of respondents	Percent
Only to mother	3	8.8
Both doctor and patient	5	14.7
Doctor, patient and family member involved	25	73.5
Only to family member involved	1	2.9
Total	34	100.0

The various problems faced by doctors because of the PNDT Act were: excessive clerical work (85.29%), administrative difficulties (44.1%), excessive police interference (29.4%), and social difficulties (8.8%) [Table 2].

**Table 2:** Different kinds of difficulties faced by doctors because of the PNDT Act*

Difficulties	No. of respondents	Percent
Excessive clerical work	29	85.29
Social difficulties	3	8.8
Hospital administrative difficulties	15	44.1
Police interference more than expected	10	29.4
No difficulties	6	17.6

* Multiple responses

## Discussion

Almost all (97%) of the doctors affirmed that there is demand for gender determination of the fetus by patients. This is quite shocking and points toward the mindset of society toward a girl child. The observations highlight the need for sensitization of the society towards the hazards of adverse gender ratios and changing their attitudes towards girl children.

Certain amendments were suggested in the PNDT Act by doctors viz. registration of sonography machines should be one time and not periodical, on changing the place of the clinic there should be no re-registration for the same sonography machine, etc. Dutta in her article also stated that radiologists were in favor of suitable amendments in the PNDT Act.(1)

A majority of the doctors felt that the penalties for violating the PNDT Act are too stringent and were in favor of liberalization in punishment for minor administrative lapses in implementation of the PNDT Act. It is suggested that while revising the Act, these suggestions may be given consideration. A majority of the doctors believed that the measures to improve the gender ratio should be multipronged. Thus, other social measures should be taken to raise the gender ratio. A majority of the doctors (73.5%) felt that apart from the doctor, the patient and family member involved in gender determination should also be punished for violation of the PNDT Act. Reportingpeople.org also mentions the speech of the former chief justice of India regarding couples who seek female feticide being punished.(2) Major difficulties incurred by doctors in the implementation of the PNDT Act(3) were: excessive clerical work (85.29%), administrative difficulties (44.1%), and excessive police interference (29.4%). Harassment of doctors of diagnostic centers by health officials in Ludhiana was reported by the Ludhiana Tribune.(4)

## Conclusions

Doctors were in favor of the PNDT Act but felt something extra needs to be done to improve the gender ratio. Observations on perception of doctors regarding the PNDT Act indicate a need for suitable amendments to this Act.
